# Firing up the Tumor Microenvironment with Nanoparticle-Based Therapies

**DOI:** 10.3390/pharmaceutics13091338

**Published:** 2021-08-26

**Authors:** Yunfeng Pan, Xueru Song, Yue Wang, Jia Wei

**Affiliations:** 1 The Comprehensive Cancer Centre of Drum Tower Hospital, Medical School of Nanjing University & Clinical Cancer Institute of Nanjing University, Nanjing 210008, China; 161232029@smail.nju.edu.cn (Y.P.); dg20350078@smail.nju.edu.cn (X.S.); wangyue2012nju@163.com (Y.W.); 2 Chemistry and Biomedicine Innovation Center (ChemBIC), Nanjing University, Nanjing 210008, China

**Keywords:** nanoparticle, immunotherapy, tumor microenvironment, cancer vaccine, adoptive cell therapy

## Abstract

Therapies mobilizing host immunity against cancer cells have profoundly improved prognosis of cancer patients. However, efficacy of immunotherapies depends on local immune conditions. The “cold” tumor, which is characterized by lacking inflamed T cells, is insensitive to immunotherapy. Current strategies of improving the “cold” tumor microenvironment are far from satisfying. Nanoparticle-based therapies provide novel inspiration in firing up the tumor microenvironment. In this review, we presented progress and limitations of conventional immunotherapies. Then, we enumerate advantages of nanoparticle-based therapies in remodeling the “cold” tumor microenvironment. Finally, we discuss the prospect of nanoparticle-based therapies in clinical application.

## 1. Introduction

Immunotherapies have already switched the pattern of cancer treatment and incredibly extended patient survival in melanoma, non-small-cell lung carcinoma (NSCLC), and gastric/gastro-esophageal junction cancer [1]. Immune checkpoint inhibitors (ICI), adoptive cell therapy (ACT), and cancer vaccines are major strategies of cancer immunotherapies. Several ICIs and ACT have been approved by Food and Drug Administration (FDA) and recommended by National Comprehensive Cancer Network (NCCN) guidelines as standard therapies for specific solid tumors and hematology neoplasms [2]. However, the overall response rate of immunotherapies is inferior, which indicates that it is necessary to screen potential beneficial patients [3]. Meanwhile, resistance to immunotherapies seems inevitable. Suppressive tumor microenvironment plays an important role in primary or secondary resistance to immunotherapy [4]. Tumor microenvironment (TME) remodeling exhibits synergism with immunotherapies [5]. According to immune cell infiltration and reactivity to immunotherapies, malignancies can be divided into “hot”, “cold”, and transitional types [6]. Tumors with “cold” immune landscapes are considered as refractory cases and resistant to immune agents. Although vast efforts have been made to improve immune cell infiltration and reverse immune suppressive TME, clinical outcomes are far from satisfying [7,8]. There is an urgent need to develop new methods to heat up TME.

The rapid development of nanotechnology in recent years brings a novel choice of immune agent carrier [9]. Nanoparticles are defined as materials, structures, devices, and systems with their size and shape in the nanoscale range (1 to 100 nm) [10]. Due to the similarity to biologic molecules in scale, nanoparticles are designed to execute different functions as medical agents. According to material, nanoparticles can be divided into lipid based nanoparticles, polymeric nanoparticles and inorganic nanoparticles [11].Nanoparticles have been envisioned as an attractive adjunctive approach to enhance immunotherapies [12,13]. Nanoparticles can deliver immunogens accurately and activate both antigen presenting cells (APC) and effector cells to enhance every step of anti-tumor immune cycle [14]. Besides, multiple types of drugs have been proved to display synergistic effect with immunotherapies [15]. Nanoparticles construct a platform which facilitates the combination of different therapies. In this review, we discuss the barriers of immunotherapy and focus on the application of nanoparticles to heat up the “cold” TME from different aspects.

## 2. Features of “Cold” Tumor and Barriers for Immunotherapy

Recent advances of technology, analysis methods, and mechanisms in immunology enable more specific classification of immune landscape of tumor. Due to the relationship between immune contexture and prognosis, scientists constructed immunoscore to quantitatively evaluate immune cell infiltration in both tumor center and invasive margin [16]. The cut point between a cold tumor and hot tumor is not explicit. Thus, transitional types may be more common. According to immunoscore, Galon et al. suggested a more comprehensive main four-category classification of tumors—hot, altered-excluded, altered-immunosuppressed, and cold [17]. Beyond hot, the remaining three types of tumors exhibit cold immune features in various degrees. Cold tumors feature in barrier molecules in extracellular matrix (ECM), low immunogenicity, and low antigen presentation. Cold tumors possess intrinsic insensitivity to immunotherapy. Excluded type displays notable hypoxia and angiogenesis in the center of tumor bulk thus blocking T cell trafficking. The immunosuppressed type has relatively better T cell infiltration and striking immunosuppressive factors or cells.

**Figure 1:**
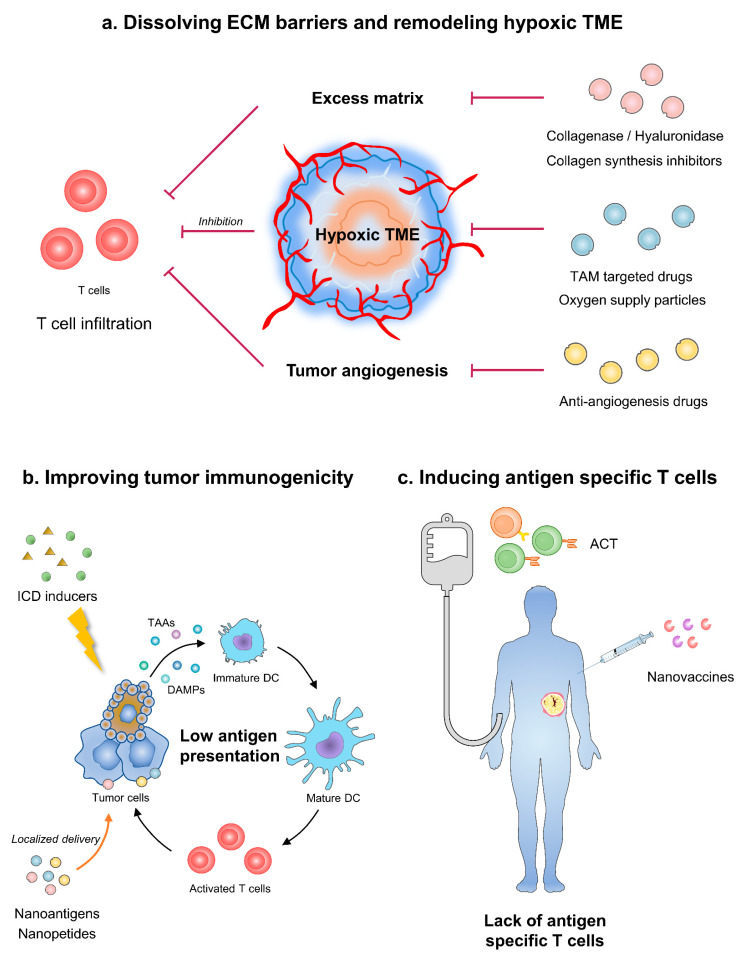
Strategies of nanoparticles in improving cancer immunotherapy. (**a**) Nanoparticles dissolving ECM barriers and remodeling angiogenesis-caused hypoxia could improve T cell infiltration. (**b**) Nanomodified ICD inducers or vaccines can augment tumor immunogenicity. (**c**) ACT equipped with nanoparticles induce antigen specific T cells and relieve systematic immune dysfunction. ECM, extracellular matrix; TME, tumor microenvironment; TAM, tumor-associated macrophage; ICD, immunogenetic cell death; TAAs, tumor-associated antigens; DC, dendritic cells; ACT, adoptive cell therapy.

Taken together, the obstacles of cold tumor immunotherapy include local immune barriers and systematic immune dysfunction. In the tumor area, molecules such as collagen and hyaluronic acid (HA) construct the ECM barrier and block immune cell infiltration [18]. Aberrant tumor vessels attenuate T cell adhesion and penetration [19]. Subsequently, tumor angiogenesis-caused hypoxia restrains the priming of immune system. Moreover, the local immunogenicity of tumor is not enough for APCs [20]. Meanwhile, cancer patients, especially in late stages, may have malnutrition and T cell exhaustion, which are systematic immune disadvantages (Figure 1).

Multifunctional nanoparticles can augment the effect of immunotherapy by resolving critical immune barriers. Nanoparticles mainly act as carriers for immunotherapy. According to distinctive design, nanoparticles are divided as tumor targeting and lymph organ targeting. Tumor-targeting nanoparticles mainly rely on the enhanced permeability and retention effect (EPR) of solid tumors [21]. Therefore, less kidney, liver, and spleen clearance also contribute to the concentration of nanoparticles in tumor bulk [22]. Tumor targeting nanoparticles provide drugs with the targetability towards TME, including ECM components, aberrant angiogenesis, hypoxia, and immune cells. Meanwhile, lymph organ targeting nanoparticles are usually administered through subcutaneous, intradermal, intramuscular, or intraperitoneal injection, which facilitates nanoparticles entering lymph circulation [23]. When reaching lymph nodes, nanoparticles with suitable diameter are absorbed by macrophages or dendritic cells and initiate antigen processing and presentation [24]. Apart from carriers, nanoparticles with multiple modification facilitate the combination of immunotherapy and other therapies. For example, nanomodification endows T cells with enhanced cytokine secretion which augments systematic antitumor immune.

## 3. Dissolving ECM Barriers

A large amount of ECM is one of the features of cold tumors [25]. Ingredients of tumor ECM, such as collagen and HA, compose physical barriers for lymphocyte infiltration or immune agents entering TME [26]. Researchers have already developed several ways to break through the ECM barriers. It is an effective strategy to break through ECM barrier by directly decomposing matrix components through nanomaterials. In addition, nanomaterials can also improve matrix properties and enhance immune infiltration by regulating stromal cells. Conventional preparation caused relatively short half-life and less-than-effective local concentration when systematically injected. Nanoparticles as carriers for those drugs avoid inappropriate activation in peripheral blood and release drugs accurately in TME.

### 3.1. Enzymolysis of Collagen and HA

Enzymes that can dissolve collagen or HA have been under trial [27]. Methods to decompose collagen could modulate tumor ECM, which could potentially increase immune cell infiltration and tumor cell evasion simultaneously [28]. Therefore, collagenase is usually applied as combination instead of monotherapy. Collagenase modification on the surface of pegylated gold nanoparticles could increase tumor penetration by 35% in the NSCLC xenograft murine model [29]. In the breast cancer murine model, collagenase conjugated with gold-nanoparticles can also improve the penetration of metformin gold-nanoparticles’ conjugation, thus reforming tumor suppression [30].

When combined with chemotherapy, collagenase could raise drug penetration and immunogenetic cell death (ICD). Fibrosis in ECM is typical of ductal adenocarcinoma of pancreas (PDAC), which has little inflamed T cells [31]. Zinger et al. developed nano-liposome encapsulated collagenase type-I called “Collagozome” [32]. Pretreatment with Collagozome followed by paclitaxel micelles decreased tumor volume by 60% in orthotopic PDAC murine model, comparing with unmodified collagenase pretreatment. Masson’s trichrome histological staining confirmed that the collagen level was 37% less in the Collagozome-treated group than free collagenase. Besides, the application of Collagozome did not increase the amount of circulating tumor cells or metastasis. Improved tumor suppression of paclitaxel micelles was in line with a higher level of ICD. It has been proven that paclitaxel-induced ICD could promote antigen presentation by dendritic cells and activate antitumor immunity [33]. Instead of utilizing collagenase as pretreatment, Huang et al. designed a novel collagenase IV and clusterin modified polycaprolactone-polyethylene glycol (PCL-PEG) nanoparticles that load doxorubicin, which exhibited impressive ECM penetration and anti-tumor effects both in vitro and in vivo [34].

HA is another druggable target of tumor ECM. The most frequently applied hyaluronidase is pegylated recombinant human hyaluronidase (PEGPH20). PEGPH20 monotherapy could increase NK cell infiltration in the high-HA tumor model [35]. Besides, PEGPH20 has been combined with chemotherapies and immunotherapies in various trials. However, the effect of nanoparticle as carriers for hyaluronidase was more controversial in clinical trials, especially combined with chemotherapies. In SWOG S1313, a phase I b /II trial, PEGPH20 plus FOLFIRINOX caused more adverse events, reduced treatment duration, and seemed to be deleterious in unselected metastasis pancreas cancer (mPC) patients [36]. While in the HALO-202 trial, retrospective analysis showed that the PEGPH20 plus nab-paclitaxel/gemcitabine group had a better objective response rate (ORR, 45% vs. 31%) and medium overall survival (OS, 11.5 vs. 8.5 months) in HA-high untreated PDAC patients [37]. Thus, the following phase III clinical trial HALO-301 was limited to high-HA PDAC patients. Considering the similar clinical background of SWOG S1313 and HALO-202, the divergence of outcomes lay in the type of chemotherapy. Patients in the PEGPH20-treated group tended to have more adverse events, although not reaching statistical significance in HALO-202 trial [38]. When combined with intense chemotherapy, enhanced adverse events may be intolerable and result in relatively more drop-outs. Taken together, caution should be paid when combing nanoparticle modified hyaluronidase with chemotherapy.

Immunotherapy is a better choice to combine with hyaluronidase, which could directly increase T cell infiltration or improve the concentration of immune agents in TME. Blair et al. applied an irradiated whole-cell PDAC vaccine along with PEGPH20 in metastasis PDAC murine model, resulting in increased effector memory T cell infiltration, IFNγ secretion, and improved survival [39]. Apart from the vaccine, hyaluronidase coordinated with the immune checkpoint inhibitor as well. PEGPH20 sensitized HA accumulating cancer to PD-L1 blockade in the breast cancer murine model [40].

Overall, nanoparticle-modified enzymolysis of collagen and HA itself enhanced immune infiltration and exhibited a synergistic effect with other immunotherapy. Enzymolysis of ECM ingredients may be potential adjuvant of immunotherapy.

### 3.2. Reprogramming ECM Producing Cells

Aside from directly dissolving ECM components, the strategy of restraining fibroblasts from secreting excessive stroma is another way of remodeling tumor ECM for better immune infiltration. Angiotensin receptor II blockers, namely losartan, inhibit collagen I synthesis in cancer-associated fibroblasts and facilitate the distribution and efficacy of pegylated liposomal doxorubicin in murine breast, pancreas, and skin cancer models [41]. A phase II clinical trial further confirmed that in neoadjuvant setting, FOLFIRINOX along with losartan brought about survival benefits to PDAC patients with an R0 resection rate of 61% [42]. Considering the regulation of TGFβ signaling of losartan, the drug may have undefine influence on anticancer immune [43]. Hou et al. developed transformable nano assemblies of carbon dots containing doxorubicin and Fe ions on the surface and losartan encapsuled within the mesopores, which exhibited about 2.40-fold higher CD8^+^ and CD4^+^ T cell infiltration than those of control [44]. Another angiotensin II type 1 receptor blocker telmisartan inhibits the development of transient hypoxia and sensitizes tumor to radiation, causing higher level of ICD [45].

Taken together, nanoparticles as carriers for ECM targeting therapies have exhibited impressive improvement of pharmacokinetics, either in prevention from premature emission or systematic toxicities. Nanoparticles when combined with chemotherapies increase T cell infiltration by inducing higher levels of ICD. Immune agents modified with nanoparticles could remodel TME more directly through better penetration and raised local concentration.

## 4. Remodeling Tumor Angiogenesis Induced Hypoxic Microenvironment

Aberrant angiogenesis is one of the hallmarks of cancer [46]. Abnormal tumor vasculatures prevent immune cells from adhesion and subsequent penetration, leading to insufficient T cell infiltration in TME [19]. Improving penetrability of T cells and normalization of tumor vasculatures can heat up cold TME [47]. Due to aberrant tumor vasculatures, hypoxia is prevalent in tumor bulk and favors the construction of immunosuppressive TME [48]. Hypoxia generally appears along with the accumulation of metabolic byproducts and immunosuppressive modulators [49]. Hypoxia markedly prevents the infiltration of effector immune cells, creating a relatively “cold” immune microenvironment [50]. Meanwhile, hypoxia induces the expression of immune checkpoints, such as PD-1, CD47 [51]. Thus, improvement of oxygen-deficiency could also heat up the immune system in the tumor area. Strategies targeting tumor angiogenesis-induced hypoxic microenvironments include the direct blockade of angiogenesis factors, regulation of the cells related to angiogenesis, and reversal of oxygen deficit.

### 4.1. Targeting Angiogenesis Factors

Nanoparticles modified anti-angiogenesis drugs exhibit better stability, targetability, and minor systematic toxicities. Song et al. developed PEG and mannose-based nanoparticles modified with trimethyl chitosan and citraconic anhydride grafted polyallylamine hydrochloride (PEG = MT/PC NPs), delivering VEGF/placental growth factor (PIGF) siRNA to both cancer cells and M2-TAMs, which normalized tumor vascular, repolarized TAMs, and suppressed tumor progression simultaneously [52]. Besides, nanoparticles as carriers for anti-angiogenesis therapies facilitate the combination with other immune agents. In several preclinical trials, the combination of ICIs and low-dose anti-angiogenesis therapies displayed synergetic effects [53]. RGD-modified lipid nanoparticles carrying VEGFR2 siRNA combined with PD-1 monoclonal antibody increased CD8+ T cell infiltration by 2–3 fold compared with single therapy [54]. Nanoparticles as carriers for anti-angiogenesis therapies provide more selectable ways to inhibit the VEGF pathway, siRNA etc. The combination of nano-modified anti-angiogenetic agents and immune therapy has synergetic effects, which deserve exploration in depth.

### 4.2. Regulating Angiogenesis Related Cells

TAMs, especially M2 phenotype macrophages, are highly related to tumor angiogenesis [55]. Therefore, nanoparticles targeting TAMs may potentially normalize tumor angiogenesis and improve hypoxia indirectly [56]. Saccharides can be recognized by macrophage mannose receptor (MMR) on TAMs. Saccharides modification provides nanoparticles with targeting effect towards TAMs. Zang et al. constructed lipid-coated mannose-modified nanoparticles as carriers for calcium zoledronate, which reduced tumor angiogenesis and remodeled immunosuppressive TME [57]. Doxorubicin hydrochloride-loaded nanoparticles modified with zymosan (ChiNPs), developed by Pawar et al., could switch TAM polarization towards the M1 phenotype and reduce VEGFR2 expression in TME [58]. PLGA nanoparticles encapsulating melanoma antigen Hgp peptide and M2-targeting peptides on the surface successfully transformed M2-like TAMs to M1 phenotype, thus normalizing tumor angiogenesis and increasing CD8^+^ T cells and NK cell infiltration [59].

Apart from directly targeting macrophage, several nanoparticles have been developed as drug carriers to target hypoxia sites, which also displayed regulation of TAMs. Carbonic anhydrase IX (CA IX) directed nanoparticles containing apoptosis inducer, revealed remarkable tumor core penetration accompanied with repolarization of TAMs [60]. Moreover, under ischemia circumstances, the homing of stem cells relies on the chemokine SDF-1α and its receptor CXCR4 [61]. Jiang et al. targeted tumor hypoxia with nanoparticle-engineered CXCR4-overexpressing adipose-derived stem cells [62]. Hypoxia-activated chemicals such as tirapazamine (TPZ) when modified with PEG-PCL have been proven to trigger ICD and boost dendritic cell (DC) maturation, subsequently activating toxic T lymphocytes [63]. Nanoscale metal-organic frameworks have emerged as unique carriers for immunoadjuvants by generation of reactive oxygen species (ROS) for ICD and in situ cancer vaccination [64].

Through the regulation of angiogenesis-related cells, nanoparticles enhanced immune cell infiltration and heated up TME. Combining these particles with ICI or other immune agents has the potential to comprehensively remodel immune suppressive microenvironments.

### 4.3. Improving Oxygen-Deficiency

Nanoparticle-constructed platforms could deliver oxygen compounds to hypoxia microenvironments and release oxygen there, thus breaking the barrier of oxygen deficit. The hollow manganese dioxide (H-MnO_2_) nano-platform encapsuled with DOX and photodynamic agent chlorine e6 relieved tumor hypoxia and increased CD8^+^ effector T cell infiltration [65]. Hybrid protein oxygen nanocarrier with chlorine e6 encapsulated (C@HPOC), markedly relieved tumor hypoxia and enhanced infiltration of CD8^+^ T cells and ICD in tumors [66]. Apart from directly delivering oxygen to TME, strategy of reducing oxygen consumption is another direction under exploration. Yang et al. has developed lase-activated PEG-PCL liposomes containing IR780 and metformin which both constrains mitochondrial respiration locally [67]. PEG-PCL liposomes combined mitochondria-targeted photodynamic therapy and photothermic therapy, thus extending the survival of MKN-45 bearing nude mice. Nano-modified hypoxia targeting agents raises the concentration of oxygen in TME, thus improving the effect of T cells.

Hypoxia is one of the core mechanisms of resistance to immunotherapies [68]. Hypoxia targeting nano-therapies resensitize tumors to immune therapies. PLGA encapsulating water-soluble catalase (Cat) and hydrophobic imiquimod, a Toll-like-receptor-7 agonist, can greatly enhance radiotherapy efficacy and displayed synergism with CTLA-4 blockade [69]. Liposomes encapsuling CAT, H_2_O_2_, and CTLA-4 antibody improved infiltration of macrophages and T cells, while reducing the percentage of M2 macrophages [70]. Liu et al. fabricated Mn@CaCO3/ICG nanoparticles loading PD-L1 siRNA, which released oxygen in the tumor site, enhanced the efficacy of photodynamic therapy, and subsequently remodeled immunosuppressive TME [71]. Application of the hypoxia-activated prodrug TH-302 sensitized typical “cold” tumor, prostate cancer, to immune checkpoint inhibitors [50]. Hypoxia is a common feature of TME, which participates in undesirable immune cell infiltration, accumulation of immune suppressive factors, and resistance to immunotherapies [68]. Nanotechnology has provided a broader horizon for developing innovative anti-hypoxia therapies.

Remodeling tumor angiogenesis-induced hypoxic microenvironments with nanomaterials has revealed valid effect on heating up cold TME. However, more efforts should be made to accomplish the translation to clinical scenarios.

## 5. Improving Tumor Immunogenicity

Efficient antitumor strategies must fully activate endogenous tumor immunity. However, one of the major obstacles to tumor clearance is loss of tumor antigen expression and low adjuvancity [72]. To solve this problem, increasing efforts have been made to improve tumor immunogenicity by enhancing local antigen presentation or adjuvanticity and inducing ICD, in order to form the cycle of immune priming, tumor cell death, antigen release, and immune reactivating, to maximize the anti-tumor effect. Nanomaterials play an important role in antigen delivery and ICD induction.

### 5.1. Assisting in Exogenous Antigen Delivery

Effector T cells recognize and bind the peptide-MHC complex on target cells to start the killing process. Lacking of endogenously presented antigen-derived peptides on tumor cells is one of the important reasons for forming “cold” TME. Therefore, the innovative approach to modify tumor cells via immunogenic antigen or peptide delivery can be an option to induce the cytotoxic T lymphocyte (CTL)-mediated antitumor activity.

Delivering alloantigens to “foreignize” tumor cells is a feasible way to enhance the immunogenicity of tumor. A nanoplatform of hyaluronic acid, which modified CD44^+^ tumor-targeting ligand and loaded with foreign antigen ovalbumin (OVA), realized preferential aggregation on tumor surface, phagocytosis by tumor cells, degradation by hyaluromycin, and release of OVA intracellularly. The OVA was degraded by proteasome into recognized peptide to boost T cell response [73]. In addition, based on the characteristics of acid pH, hypoxia, high levels of MMP, and other specific enzymes of TME, the stimulation sensitive nanodelivery system can improve the safety of the antigen delivery therapy. For example, a conjugated polymer nanoplatform modified by matrix metalloproteinase 9 (MMP9) cleavable linker allowed foreign antigen to be delivered and conditionally released into the local tumor site [74]. This kind of exogenous antigen-loading therapy theoretically overcomes the tumor heterogeneity and has a certain clinical application potential. However, the current preclinical studies are mostly the introduction of heterologous proteins, and its safety needs to be further evaluated.

Delivering viral peptides can also subtly enhance the immunogenicity of tumor cells. Memory T cells specific to previous virus infections can produce immediate and effective response to secondary infection. Injecting non-replicating viral peptides into TME effectively reactivated these antiviral T cells by mimicking a viral reinfection to poorly immunogenic tumor cells [75]. Encouragingly, antibody-peptide epitope conjugates (APECs), a nanoscale antibody and viral peptide conjugates, successfully loaded CMV antigens to the tumor surface, mobilized pre-stored virus specific memory T cells to attack tumor cells, effectively increasing the immunogenicity of tumor cells and avoiding the potential biosafety problem of oncolytic virus infection [76]. Redirection of virus-specific T cells to tumors may yield new therapeutic opportunities for cancer patients. However, previous reports mainly focused on intratumoral injection. It is only suitable for superficial and puncture accessible lesions, rather than microsatellite lesions and metastatic lesions. Therefore, it is necessary to develop effective tumor targeting and penetrable materials for systemic administration in order to eliminate occult lesions.

### 5.2. Promoting the Release of Endogenous Antigen

Apoptotic tumor cells induced by subtherapeutic doses of chemotherapeutics, radiotherapy, or photodynamic therapy could release tumor associated antigens (TAA), damage-associated molecular patterns (DAMPs) and pro-inflammatory cytokines to trigger antitumor immune response, which called ICD [77]. ICD-inducing modalities can effectively provoke specific T cell responses while killing tumors, and eventually transform a “cold” TME to an immunogenic, “hot” TME [78].

The application of the nanomaterials endows ICD inducers with superior antitumor activity. Apart from being a synergist of ICD to improve penetration and hypoxia microenvironment as above-mentioned, nanoparticles have several unique advantages in inducing ICD. First, aggregation in tumor site is necessary for ICD inducers. Integrated mesoporous silica nanoparticles armed with classical ICD inductors doxorubicin (DOX), named DOX@HIMSNs, initiated an anti-tumor immune response characterized by DC maturation and antitumor cytokines release [79]. Second, nanotechnology allows ICD inducers to release in a predictable and designable manner. DOX@HIMSNs has been confirmed to mostly accumulate in tumor tissue and controllably release DOX in acidic microenvironment with high concentration of GSH with the help of integrating a pH and GSH dual stimulated rotaxane [79]. Third, nanoparticles could effectively induce ICD while reducing their side effect. NPs can selectively deliver photosensitizers to tumors with minimize damage to normal tissues by spatially controlled light irradiation [78]. Fourth, co-loading multi-components on nanoparticles could significantly improve ICD and anti-tumor effect. Sen et al. engineered a redox-active Au(I) bis-N-heterocyclic carbine (Au(I) bis-NHC) that realized the double effect combining TrxR2 inhibition (damaging biological antioxidants) with increased oxidative stress [80]. The combination of photodynamic therapy with oxygen therapy based on C@HPOC showed enhanced specific CD8^+^ T cell response and abscopal effect [66]. Fifth, the development of nanoparticles broadens the selection range of ICD inducers. Classical metallic ICD inducer oxaliplatin failed to induce ICD in non-small cell lung cancer (NSCLC) [81]. An ER-targeting iridium(III) complex, armed with an N,N-bis(2-chloroethyl)-azane derivate, significantly triggered endoplasmic reticulum stress and increased reactive oxygen species by targeting endoplasmic reticulum, resulting in antitumor CD8^+^ T cell response and Foxp3^+^ T cell depletion, successfully suppling the selection of ICD inducers for NSCLC [82]. At last, nanoparticles can be used as a synergist of ICD inducers. Min et al. engineered Antigen-capturing NPs (AC-NPs) could play a good synergy with radiotherapy by capturing TAAs released after radiation with different surface chemistry and transport them to APCs [83].

## 6. Inducing Antigen Specific T Cells

The essence of cold tumors is lack of a pre-existing immune response [17]. Vaccine-base approaches, which include therapeutic vaccines and ACT, are the gateway to overcome failed spontaneous T cell priming by inducing the activation and expansion of specific T cells in vivo or infusing engineered T cells.

### 6.1. Enhancing Therapeutic Vaccines

Therapeutic vaccines, consisting of antigens (usually provided in DNA, RNA, full proteins, peptides, or even whole tumor cells) and adjuvants, aim to prime the antigen presentation process of DCs, activate and expend tumor-specific T cells, and eventually lead to tumor killing specifically [84]. In addition to the above-mentioned methods to improve local immune infiltration, vaccine-based immunotherapy that enhances systemic T cell responses is a promising approach to overcome the lack of a pre-existing immune response, and ultimately fire up the TME. Traditional cancer vaccines such as tumor-associated antigens (TAA) exhibited no better efficacy than standard therapies in most clinical trials due in large part to central tolerance and low TCR binding affinity [85]. With the development of biological detection, neoantigens produced by gene mutation, which are not expressed in normal tissues and theoretically have no central tolerance, are the most attractive vaccine targets in recent years. The clinical trials on glioblastoma, which is typically an immunologically “cold” tumor, proved that personalized neoantigen vaccines would promote neoantigen-specific T cell amplification and increase the number of tumor infiltrating T cells [86,87]. In addition to appropriate epitope selection, efficient delivery has always been an urgent problem in the field of therapeutic vaccines. Nanomaterials can significantly improve the delivery and therapeutic efficacy of vaccines through multiple epitope loading, preventing degradation, targeting and retention effects.

The antitumor immune responses mainly take place in secondary lymphoid organs, such as draining lymph nodes. With the application of nanomaterials, antigens and adjuvants can be well enriched and precisely released in lymph nodes. For example, a bi-adjuvant nanovaccine-carrying neoantigen significantly activated DCs and prolonged neoantigen presentation compared with soluble peptide and adjuvants [88]. The nanomaterials as carriers include liposomes [89], inorganic nanoparticles [90], polymeric nanoparticles [91], nanogels [92], nano-nucleic acid [93], self-assembled protein [94], and so on. The size and surface characteristics of nanovaccines determine their lymph node delivery efficiency and DCs regulation. After subcutaneous administration, medium-sized (5 to 100 nm) nanoparticles are appropriate for drainage and retaining in the lymph nodes [88]. The positively charged nanoparticles are more easily captured by antigen presenting cells, because the extracellular matrix is composed of collagen fibers and negatively charged proteins such as glycosaminoglycans [95]. In addition, the surface modification of nanovaccine delivery system can enhance its lymph node targeting and achieve more accurate drug delivery. Studies have proven that DC-targeting ligands, such as CD11c, CD40, Dec205, and so on, could be modified on nanovaccines and then improve DCs uptake [84]. Recently, the concepts of magnetic targeting, pH targeting, thermal targeting, and enzyme targeting are gradually being applied in the delivery of nanovaccines. Notably, a recent study in a mouse model have demonstrated that an intravenous nanovaccine would generate more stem-like neoantigen-specific CD8^+^ T cells, leading to a superior antitumor response, and thus suggesting that the route and dose is important in optimizing antitumor immunity as well [96]. In a first-in-man phase I clinical trial, DPX-9701, a liposome modified peptide vaccine was exhibited enough immunogenicity and resulted in a 61% immunological response rate [97]. Besides, other nano-modified vaccines have exhibit hopeful effects in several clinical trials (Table 1). Therefore, nanovaccines have vast clinical prospects.

Particles larger than 500 nm could not enter the lymphatic reflux system and remained in the extracellular matrix [98]. Based on this principle, three-dimensional porous scaffolds have been developed, which recruit a large number of immature DCs, and release antigens and adjuvants to reprogram DCs [84]. As an example, mesoporous silica rod-based vaccines serving as a powerful multi-antigen platform realized excellent tumor regression [99]. Compared with traditional nanovaccines, this in situ process avoids the risk of the nanomaterial retaining in non-immune organs. However, the optimizing controllable release, injectability, and degradability for scaffold-based vaccines remains to be further explored.

### 6.2. Optimizing Adoptive Cell Therapy

Adoptive cell therapy (ACT), which refers to transfusion of antigen-specific T cells such as tumor infiltrating lymphocytes (TILs), cytotoxic T lymphocytes (CTLs), chimeric antigen receptor (CAR) T cells, T cell receptor (TCR) T cells, and so on, has shown remarkable clinical success in leukemia and melanoma [92]. However, several obstacles, which include lack of suitable targets, suboptimal T cell specificity or activation, inhibitory immune microenvironment, abnormal blood vessels, and dense extracellular matrix, limit the application of ACT in solid tumors. Thus, in addition to choosing appropriate targets, aggressive ACT regimens are needed to achieve a modest effect in solid tumors [100]. Nanotechnology has the advantages of improving the function of adoptive T cells and protecting them from being suppressed by TME.

Obtaining enough adoptive T cells is the premise of ACT. In vitro artificial antigen presenting cells (aAPC) based on nanoparticles (including antigen peptide/MHC molecular complex, costimulatory molecules, and membrane-bound cytokines, etc.) can realize the efficient activation and expansion of T cells in vitro, and lay the foundation for further T cell modification [84]. For example, a polyethylene glycol hydrogel platform, decorated with integrin-activating peptides and anti-CD3 antibodies coupled gold nanoparticles, successfully initiated integrin-mediated T cell adhesion, and expanded T cells prior to transfusion [101].

Therapeutic T cell engineering with surface-conjugated synthetic nanoparticles can effectively enhance their killing effect [102]. Nanoparticles on the surface of T cells usually release immunomodulators under specific conditions, which can enhance the function of adoptive T cells by autocrine or regulate TME by paracrine. Li et al. designed a cell surface-conjugated protein nanogel (NGs) loaded with IL-15 super-agonist complex that responded to increased reduction potential in T cell surface after antigen recognition, leading to 16-fold T cell expansion and improvement of tumor clearance [92]. Nie et al. bound the magnetic nanoclusters with pH-responsive PD-1 antibody on to effector T cells, which could realize the recruitment into TME through MRI guidance and enhance the effector function by releasing PD-1 antibody [103].

Although ACT therapies are effective strategies to eliminate cancer, they are limited by autograft, high cost and complicated manufacturing process. Yu et al. modified nontoxic naive T cells with circular bispecific aptamers (cb-aptamers) on the surface, resulting in T cells efficiently accumulating and anchoring at tumor sites. Then the engineered naive T cells were subsequently activated in situ by CD3/CD28 beads to induce tumoricidal activity [104]. This kind of “recognition-then-activation” strategy provides a new idea for making ACT therapy more universal and concise. However, the safety and activation methods of naive T cells need to be further optimized.

The premise of ACT in solid tumors is that immune cells effectively infiltrate into tumor lesions. Exploring optimal strategies to promote the infiltration of adoptive T cells in solid tumors has been an academic focus. The combination of ACT with other therapies that overcome the barriers of T cell infiltration above-mentioned can play an excellent synergistic anti-tumor effect. For example, the efficacy of CAR-T therapy has been significantly improved by photothermal ablation due to its impact of increasing blood perfusion as well as the release of antigens and proinflammatory factors [105]. How to maximize the infiltration of adoptive T cell and minimize toxicity by use of nanomaterial needs to be further explored.

## 7. Prospects

Recently, nanotechnology has inevitably made its way into immunotherapy. We consider that nanotechnology has the potential to improve the efficacy of immunotherapies by facilitating the delivery of specific combinations and schedules of ECM-targeting agents, cytotoxic agents, modified immune cells, and vaccines. When separately modified with nanoparticles, immune agents obtain improved targetability toward either tumor or immune organs. Tumor targeting nano-carriers are relatively well developed in line with the universal application of nano-modified chemotherapy in cancer treatment. However, due to the complexity of immune system, contemporary nano-modified immunotherapy focuses on a single aspect. Researchers’ comprehension of antitumor immune process deepens with time. Nano-modified immunotherapy will be more delicately and sophisticatedly designed to achieve satisfying synergism between various therapies. The application of nanotechnology in immunotherapy has broad possibilities of transition into clinical scenarios.

Apart from all the advantages of nanoparticles, potential disadvantages may occur along with the wild application of nano-modified immunotherapies. Unpredictable distribution is one of the dose-limiting factors. Due to concentration in reticuloendothelial system, several nanoparticles possess hepatotoxicity [106]. Moreover, the penetration of the blood–brain barrier causes relatively higher concentration of nanoparticles, especially when carrying cytotoxic agents in neuro system and neurotoxicity [107]. However, researchers have made efforts to shield patients from systematic toxicities. Nanoparticles with unique design possess better targetability and safety [108]. Besides, considering the unique physical characteristics of nanoparticles, they retain in plastic syringes and introduce dosage uncertainties which may compromise the accuracy of nanomedicine [109]. Wu et al. utilized surface active agent to improve solubility of nanoparticles, avoiding dose inaccuracy [110]. With the rapid development of nano therapy, how to raise the efficacy while minimizing toxicities is an inevitable issue.

## Figures and Tables

**Table 1:** Clinical trials of nanoparticles in the treatment of cancer.

Classification	Intervention	Description	Disease Type	NCT and Study Stage
ECM targeting	PEGPH20	Pegylated recombinant human hyaluronidase	PDAC	NCT02921022 NCT01959139 Phase I/II
			Solid tumor	NCT00834704 Phase I
			NSCLC Gastric cancer	NCT02563548 Phase I
Angiogenesis	M200	Volociximab in combination with liposomal doxorubicin	Ovarian cancer	NCT00635193 Phase I/II
	HAL and BF-200 ALA	Nanoscale photosensitizers	Basal cell Carcinoma	NCT02367547 Phase I/II
Vaccine	E75-PLG	PLG encapsuled HER2 vaccine	Breast cancer Lung cancer Ovarian cancer	NCT00005023 Phase I
	DPX-0907	Lipid based vaccine	Breast cancer Ovarian cancer Prostate cancer	NCT01095848 Phase I
	L-BLP25	Liposome MUC1 vaccine	Multiple myeloma	NCT01094548 Phase II
	ONT-10	Liposomal synthetic glycolipopeptide antigen	Solid tumors	NCT01556789 Phase I
	PDS0101	Liposomal HPV-16 E6/E7 multipeptide vaccine	Cervical cancer	NCT04580771 Phase II
	NY-ESO-1	Pegylated liposomal doxorubicin hydrochloride + NY-ESO-1 vaccine	Fallopian tube cancer	NCT01673217 Phase I

Abbreviations: MUC1, mucinous glycoprotein 1; PDAC, pancreatic ductal adenocarcinoma; HAL, hexyl aminolevulinate; ALA, aminolevulinic acid nano emulsion; NSCLC, non-small-cell lung cancer; PLG, polylactide-co-glycolide.

## Data Availability

Not applicable.
